# Factors Influencing the Measurement of Lysosomal Enzymes Activity in Human Cerebrospinal Fluid

**DOI:** 10.1371/journal.pone.0101453

**Published:** 2014-07-01

**Authors:** Emanuele Persichetti, Davide Chiasserini, Lucilla Parnetti, Paolo Eusebi, Silvia Paciotti, Claudia De Carlo, Michela Codini, Nicola Tambasco, Aroldo Rossi, Omar M. El. Agnaf, Paolo Calabresi, Tommaso Beccari

**Affiliations:** 1 Department of Pharmaceutical Sciences, University of Perugia, Perugia, Italy; 2 Department of Medicine, Section of Neurology, University of Perugia, Perugia, Italy; 3 Regional Health Authority of Umbria, Epidemiology Department, Perugia, Italy; 4 Department of Biochemistry, Faculty of Medicine and Health Sciences, United Arab Emirates University, Al Ain, United Arab Emirates; 5 Faculty of Medicine, King Abdulaziz University, Jeddah, Saudi Arabia; 6 Fondazione S. Lucia, IRCCS, Rome, Italy; university of alabama at birmingham, United States of America

## Abstract

Measurements of the activities of lysosomal enzymes in cerebrospinal fluid have recently been proposed as putative biomarkers for Parkinson's disease and other synucleinopathies. To define the operating procedures useful for ensuring the reliability of these measurements, we analyzed several pre-analytical factors that may influence the activity of β-glucocerebrosidase, α-mannosidase, β-mannosidase, β-galactosidase, α-fucosidase, β-hexosaminidase, cathepsin D and cathepsin E in cerebrospinal fluid. Lysosomal enzyme activities were measured by well-established fluorimetric assays in a consecutive series of patients (n = 28) with different neurological conditions, including Parkinson's disease. The precision, pre-storage and storage conditions, and freeze/thaw cycles were evaluated. All of the assays showed within- and between-run variabilities below 10%. At −20°C, only cathepsin D was stable up to 40 weeks. At −80°C, the cathepsin D, cathepsin E, and β-mannosidase activities did not change significantly up to 40 weeks, while β-glucocerebrosidase activity was stable up to 32 weeks. The β-galactosidase and α-fucosidase activities significantly increased (+54.9±38.08% after 4 weeks and +88.94±36.19% after 16 weeks, respectively). Up to four freeze/thaw cycles did not significantly affect the activities of cathepsins D and E. The β-glucocerebrosidase activity showed a slight decrease (−14.6%) after two freeze/thaw cycles. The measurement of lysosomal enzyme activities in cerebrospinal fluid is reliable and reproducible if pre-analytical factors are accurately taken into consideration. Therefore, the analytical recommendations that ensue from this study may contribute to the establishment of actual values for the activities of cerebrospinal fluid lysosomal enzymes as putative biomarkers for Parkinson's disease and other neurodegenerative disorders.

## Introduction

Lysosomes are involved in the degradation of macromolecules, and the impairment of their functionality due to a genetic lack of acidic hydrolases is the cause of lysosomal storage diseases (1). In recent years, lysosomal dysfunction has also been shown in patients with neurodegenerative disorders, i.e., Parkinson's disease (PD) (2, 3) and Alzheimer's disease (AD) (4, 5), where the accumulation of undegraded and misfolded proteins occurs. Mutations in the *GBA1* gene, which codes for the lysosomal glycohydrolase β-glucocerebrosidase (EC = 3.2.1.45), cause Gaucher's disease (6). Several reports have demonstrated that *GBA1* mutations are an important risk factor for PD (7, 8, 9, 10). Either the inhibition or down-regulation of β-glucocerebrosidase causes α-synuclein accumulation in cultured cells along with mitochondrial dysfunction (11). A self-propagating loop in which mutated β-glucocerebrosidase induces α-synuclein accumulation within the lysosomes that subsequently compromises lysosomal protein degradation has been postulated (12). As a further demonstration of a link between β-glucocerebrosidase deficiency and PD, decreased β-glucocerebrosidase activity has been described in the cerebellum and substantia nigra of PD patients, even in those without *GBA1* mutations (13). Alterations in lysosomal chaperone-mediated autophagy have been demonstrated to cause the reduction of β-glucocerebrosidase protein levels and activity in the brain of early stage PD patients, especially in brain regions showing increased α-synuclein levels (14). β-glucocerebrosidase activity has been found to be decreased in the CSF of PD patients compared to individuals affected by other neurological disorders (OND) (15). In another study that assessed the CSF β-glucocerebrosidase activity in patients with different dementia disorders, including synucleinopathy (dementia with Lewy bodies) and tauopathies (AD and frontotemporal dementia), a selective decrease was observed in patients diagnosed with dementia with Lewy bodies, while α-mannosidase (EC = 3.2.1.24) showed a marked decrease in all of the groups studied (16). Recently, increased β-galactosidase (EC = 3.2.1.23) and cathepsin E (EC = 3.4.23.34) activities and a reduced α-fucosidase (EC = 3.2.1.51) activity have been found in the CSF of PD patients compared to healthy controls, and β-glucocerebrosidase showed a trend toward reduction in the PD group (17). Most recently, significantly reduced β-glucocerebrosidase activity was found in a cohort of 71 PD patients compared to 45 neurological control patients. Interestingly, the β-glucocerebrosidase activity reduction was more pronounced in patients with an early stage of the disease (18).

The reasons for these different outcomes may be found both in the heterogeneity of the diagnostic groups studied (early vs late, *de novo* vs treated PD patients and healthy volunteers vs neurological patients as control groups) and in pre-analytical confounding factors, i.e., different conditions for the collection and storage of the CSF samples. Differences in the CSF handling and analytical procedures should play a negligible role in the total variability of these assays because all of the previously cited CSF studies were performed in a single laboratory.

Therefore, to determine the main pre-analytical sources of variability for the fluorimetric measurement of these enzymes, we systematically evaluated the impact of the within- and between-run variability, storage conditions and freeze-thaw cycles on the measured CSF activity of 6 lysosomal glycohydrolases, β-hexosaminidase (EC = 3.2.1.52), α-fucosidase, β-mannosidase (EC = 3.2.1.25), α-mannosidase, β-galactosidase and β-glucocerebrosidase, and two proteases, cathepsin D (EC = 3.4.23.5) and cathepsin E.

## Materials and Methods

### Subjects

Twenty-eight subjects were included in this study. Twelve of the subjects were diagnosed as having PD according to the United Kingdom Parkinson's Disease Society Brain Bank criteria (19), whereas the remaining 16 patients were affected by other minor neurological disorders (OND) requiring lumbar puncture (LP) for diagnostic purposes (epileptic seizures, transient global amnesia, mononeuropathy or postural instability). The demographic and clinical data are reported in table 1.

**Table 1:** Demographic and clinical details of the subjects included in the study.

Patient Groups	OND	PD
No (M/F)	18 (11/7)	12 (5/7)
Age (years)	67.46±7.70	66.83±8.22
Disease duration (years)	/	9.58±11.57
UPDRS	/	26.17±18.69
H&Y score	/	1.37±1.02
MMSE	25.64±4.90	24.00±5.42

UPDRS  =  unified Parkinson's disease rating score; H&Y  =  Hoehn and Yahr score; MMSE  =  mini mental state examination. The data are reported as the mean ± the standard deviation.

### Ethics Statement

The study was approved by the ethical committee of the Umbria region. None of the subjects included in the study presented with neurological deterioration. LP was performed on subjects in the OND group for diagnostic purposes. Patients were included only if they were able to understand the study aim and procedures. Written informed consent was obtained from all of the subjects included in this study.

### CSF collection

The LPs were performed between 8:00 and 10:00 AM. The CSF samples (10 ml) were collected in sterile polypropylene tubes, centrifuged for 10 minutes at 2,000×g, and stored in aliquots under the various conditions described below.

### Lysosomal enzyme assays

The activities of the CSF lysosomal enzymes were determined with the corresponding fluorigenic substrate and reaction buffer (Table 2). To measure the β-hexosaminidase, α-fucosidase and β-mannosidase, 10 µl of CSF were incubated with 40 µl of the substrate solution for 10 min at 37°C. In the α-mannosidase and β-galactosidase assays, 20 µl of CSF were incubated in the presence of 40 µl of the corresponding substrate solution for 3 h. The β-glucocerebrosidase was assayed by incubating 20 µl of CSF with 40 µl of the substrate solution with a pH = 5.0 in the presence of 0.2% taurodeoxicolic acid (TDC) for 3 h. The reactions were stopped by adding ice-cold 0.2 M glycine-NaOH buffer with a pH = 10.4 to a final volume of 0.3 ml. The fluorescence of the liberated 4-methylumbelliferone was measured on a BMG LabtechFLUOstar OPTIMA fluorometer (excitation wavelength = 360 nm; emission wavelength = 446 nm).

**Table 2:** Enzymatic assays description.

Enzyme	Substrates	Concentration (mM)	Reaction buffers	Substrate specificity
β-glucocerebrosidase	4-methylumbelliferyl-β-D-glucopyranoside	10.0	citrate/phosphate, pH 5.0, 0.2% sodium taurodeoxicolate	100%
α-mannosidase	4-methylumbelliferyl-α-D-mannopyranoside	3.0	0.2 M sodium acetate, pH 4.5	95% lysosomal α-mannosidase (5% neutral α-mannosidase)
β-mannosidase	4-methylumbelliferyl-β-D-mannopyranoside	3.0	citrate/phosphate, pH 4.5	100%
β-hexosaminidase	4-methylumbelliferyl-2-acetamido-2-deoxy-β-D-glucopyranoside	3.0	citrate/phosphate, pH 4.5	100%
β-galactosidase	4-methylumbelliferyl-β-D-galactoside	3.0	citrate/phosphate, pH 4.5	100%
α-fucosidase	4-methylumbelliferyl-α-D-fucoside	3.0	citrate/phosphate, pH 4.5	100%
cathepsin D and cathepsin E	MOCAc-Gly-Lys-Pro-Ile-Leu-Phe-Phe-Arg-Leu-Lys (Dnp)-D-Arg-NH2	0.03	50 mM sodium acetate, pH 4.0 (w or w/o 15 µM pepstatin A)	cathepsin D activity was determined subtracting the value obtained in the presence of Pepstatin A (cathepsin E activity) from the value in absence of inhibitor (cathepsin D + cathepsin E)

The specific substrate and reaction conditions are reported for each analyzed lysosomal enzyme. Sample volumes, temperature and time of incubation are indicated in Material and Methods.

The cathepsin D and cathepsin E activities were determined using MOCAc-Gly-Lys-Pro-Ile-Leu-Phe-Phe-Arg-Leu-Lys(Dnp)-D-Arg-NH_2_ as a substrate dissolved in 50 mM sodium acetate buffer with a pH = 4.0. The samples were incubated with the substrate at a concentration of 30 µM for 1 h at 40°C in the presence and absence of the cathepsin D inhibitor pepstatin A (15 µM). The reactions were stopped by adding 5% (w/v) trichloroacetic acid to a final volume of 3 ml and were measured with a Jasco FP-750 spectrofluorometer at an excitation wavelength of 328 nm and an emission wavelength of 393 nm. The value obtained in the presence of pepstatin A corresponded to the cathepsin E activity. The cathepsin D activity was calculated by subtracting the value obtained in the presence of pepstatin A from that measured in the absence of the inhibitor.

The standard curves have been created using serial dilutions of the fluorochromes 4-methylumbelliferone and Mca-Pro-Leu-OH peptide (for cathepsins). In order to minimize the standard curves variability, they have been created by the same operator using the same stock reagents solutions. Moreover, substrates were prepared and frozen into aliquots, so that in every assay the substrate was used after one freeze/thaw cycle.

One unit (U) of enzyme activity was defined as the amount of enzyme that hydrolyses 1 µmol of substrate/min at 37°C. All of the activities were measured in triplicate. All of the samples included in this study were assayed for the activities of the lysosomal enzymes within 30 min from the collection (fresh CSF). The remaining volume of each sample was used to assess the confounding factors.

### Statistical analysis

The statistical analysis was carried out with R v.2.15 (20). The descriptive statistics are presented as the mean ± SD for the continuous variables and as percentages for the categorical variables. To analyze the variability of the enzymes' activities, mixed-effects regression models were used for taking into account the repeated measurements of samples between storage techniques, freeze-thaw cycles and time points (21). The statistically significant level is considered as p<0.05.

## Results

Analysis of the effect of the diagnosis on pre-analytical factors has been performed, showing no significant differences between OND and PD groups for the measured enzyme activities (data not shown). Therefore, the results are presented considering the whole group of samples independently from the diagnosis.

### Precision of the assays for evaluating the activities of lysosomal enzymes in CSF

Eight CSF samples were flash-frozen in liquid nitrogen in ten separate aliquots. One aliquot was thawed and used for enzymatic assays each day for ten consecutive days. Each enzyme was tested in duplicate while each sample was measured in triplicate. The within- and between-run assay coefficients of variation (CVs) were calculated according to guideline EP5-A2 from the National Committee for Clinical Laboratory Standards (NCCLS) (22). As an internal control, a pooled CSF sample was run in each experiment to ensure that the measured samples were within an acceptable range, which was defined as ±2 times the standard deviation (SD) from five consecutive measurements of the same pool.

The values of the within- and between-run CV for each enzymatic assay are reported in table 3. The within-run CVs were in the range of 3.31–7.43%, while the range for the between-run variability was 5.21–9.21%. Globally, the enzyme that showed the highest total variability was cathepsin E (16.64%), while the one with the lowest total variability was β-hexosaminidase (8.63%).

**Table 3:** Within- and between-run variabilities of the activities of CSF lysosomal enzymes.

Enzyme	Within-assay CV (%)	Between-assay CV (%)	Total variability (%)
α-mannosidase	6.83	7.74	14.57
α-fucosidase	6.44	5.21	11.65
β-mannosidase	6.06	7.56	13.62
β-galactosidase	4.87	7.31	12.18
β-glucocerebrosidase	4.89	6.72	11.61
β-hexosaminidase	3.31	5.32	8.63
cathepsin D	4.36	6.06	10.42
cathepsin E	7.43	9.21	16.64

The within-assay and the between-assay coefficients of variation (CVs) together with the total variability are reported for each enzyme. The CSF aliquots stored at -80°C were measured across a ten day period.

### Ranges of the activity values for lysosomal enzymes in fresh CSF

Table 4 reports the median and the ranges of the enzymatic activities studied in fresh centrifuged CSF for all of the subjects enrolled in the study. The highest median activity was reported for β-hexosaminidase (3811.63, range = 1745.40–6919.98 mU/L), while β-glucocerebrosidase had the lowest median activity (6.52, range = 1.46–15.20 mU/L).

**Table 4:** Median and ranges of lysosomal enzymes activity in fresh CSF.

Enzyme	Median (range) (mU/L)	25–75 percentile (mU/L)
α-fucosidase	626.40 (173.25–883.92)	392.74–681.23
α-mannosidase	172.98 (7.37–250.44)	140.3–204.86
β-mannosidase	651.13 (204.63–1158.84)	388.07–747.78
β-galactosidase	11.39 (4.06–18.67)	8.29–14.89
GCase	6.52 (1.46–15.20)	4.06–7.86
Cathepsin D	516.85 (324.76–776.65)	426.93–595.62
Cathepsin E	14.54 (5.24–33.17)	10.49–18.24
β-hexosaminidase	3811.63 (1745.40–6919.98)	2980.07–4614.60

Median, ranges, 25-75% percentiles are reported of the enzymatic activities tested in fresh CSF for all the patients included in the study (n = 28). Enzyme activity was measured within 30 minutes from the lumbar puncture.

### Variability related to the freezing conditions

A previous study found an increased stability in the activities of lysosomal enzymes when the CSF was frozen in liquid nitrogen (23). To test which freezing conditions should be recommended for the measurement of the activities of lysosomal enzymes, three conditions were compared: flash freezing in liquid nitrogen, direct freezing at −80°C and direct freezing at −20°C. Twelve CSF samples were aliquoted, placed on ice and frozen within 30 min after the lumbar puncture by placing them at −20°C, −80°C or in liquid nitrogen and then stored at −80°C. Within 3 days, the aliquots were thawed on ice and assayed.

**Figure 1:**
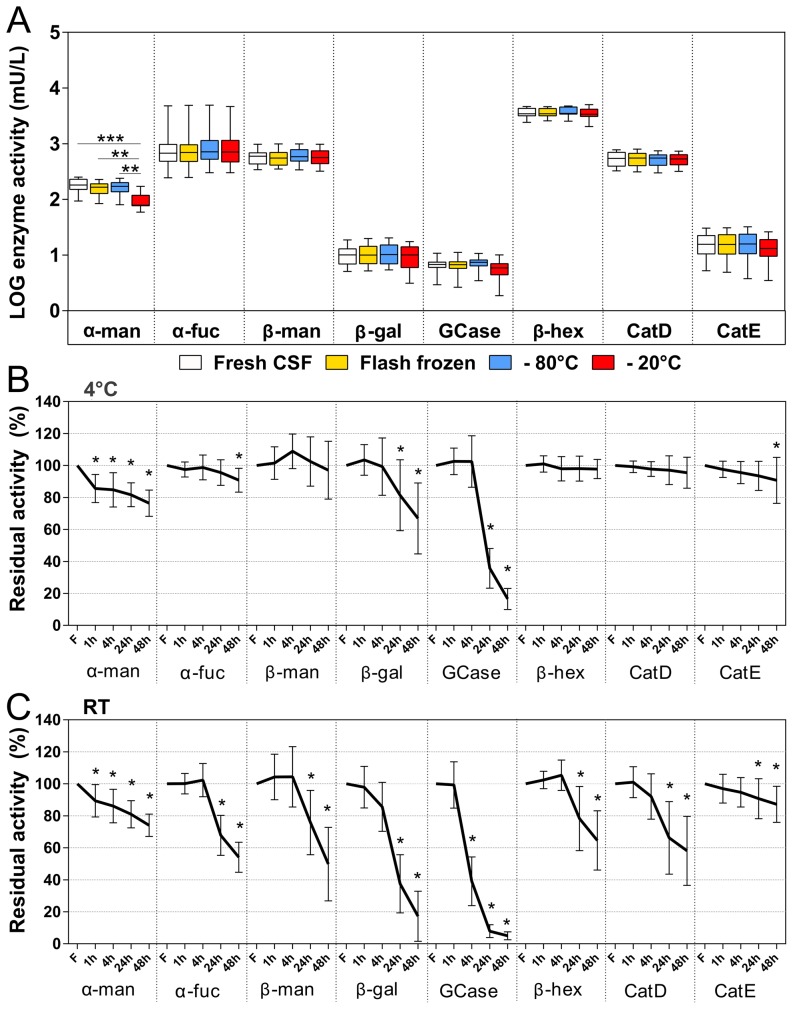
Assessment of the pre-storage confounding factors. (A) CSF samples were frozen under different conditions. Box-plots report the median activity, 25–75 percentiles and ranges. * = p<0.05; ** = p<0.01; *** = p<0.001. (B,C) CSF aliquots were kept at 4°C (B) or 22°C (C) for 1, 4, 24 or 48 hours before freezing. The results are reported as the mean percentage activity change ± the SD with respect to fresh CSF. * = at least p<0.05. α-man: α-mannosidase; α–fuc: α-fucosidase; β-man: β-mannosidase; β-gal: β-galactosidase; GCase: β-glucocerebrosidase; β-hex: β-hexosaminidase; CatD: cathepsin D; CatE: cathepsin E.

Analysis of the variance among the three conditions tested showed that only the α-mannosidase activity was significantly reduced when the sample was frozen at −20°C compared to the fresh sample (p<0.001), flash freezing the sample in liquid nitrogen and directly freezing the sample at −80°C (p<0.01). The activities of all of the other enzymes showed no significant differences among the three freezing conditions (Fig. 1A).

### Pre-storage conditions: the time delay before freezing and the temperature

Several pre-storage conditions, resembling the most common procedures for CSF storage before freezing, were tested and compared to the respective fresh CSF sample. Twelve CSF samples were divided into aliquots, which were kept at 4°C or RT (22°C). One aliquot of each group was flash-frozen after 1, 4, 24, and 48 hours and stored at −80°C. After 1 week from the last freezing, all the aliquots were thawed on ice and used to determine the activities of the lysosomal enzymes. The activity of each enzyme in each sample was compared to that of the respective fresh sample.

Fig. 1B shows the effect of storage at 4°C before freezing for time delays up to 48 hours. The most stable enzymes were β-hexosaminidase, β-mannosidase and cathepsin D, which showed no significant changes when stored at 4°C for up to 48 hours. Alpha-fucosidase and cathepsin E showed no significant changes when stored at 4°C for up to 24 hours. On the other hand, α-mannosidase was unstable at any time point. The β-glucocerebrosidase activity did not show any significant change until 4 hours of storage at 4°C, and the enzyme activity had an average decrease of −64.34±12.39% after 24 hours and of −84.88±7.85% after 48 hours (Table 5). The effect of the storage at room temperature before freezing was more evident for all of the enzymatic activities tested (Fig. 1C and Table 5). The enzymes that were most affected by the storage at room temperature were β-glucocerebrosidase and α-mannosidase, and after 4 hours, they showed decreases in their activities of −60.92±15.21% and −13.94±10.53%, respectively (Fig. 1C).

**Table 5:** Relative percentage change of lysosomal enzymes activities under different pre-storage conditions.

*Enzyme*	*T*	*1 h*	*4 h*	*24 h*	*48 h*
α-mannosidase	4°C	−14.39±8.76**	−15.25±10.75**	−18.25±7.46**	−23.57±8.17**
α-fucosidase		−2.53±4.75	−1.23±7.79	−4.4±7.98	−9.19±7.46*
β-mannosidase		1.53±10.18	8.88±10.78	2.47±15.42	−2.86±18.12
β-galactosidase		3.52±9.57	−0.71±17.93	−18.59±22.21*	−33.09±22.15**
GCase		2.56±8.27	2.51±16.07	−64.34±12.39***	−84.88±7.85***
β-hexosaminidase		1.04±5.11	−2.05±7.62	−1.93±7.81	−2.19±5.96
cathepsin D		−0.77±3.57	−2.15±4.62	−2.87±9.02	−4.52±9.73
cathepsin E		−2.37±5.09	−4.38±6.93	−6.51±9.05	−9.29±14.41*
α-mannosidase	RT	−10.59±10.13*	−13.94±10.53**	−18.96±8.49**	−25.92±6.99**
α-fucosidase		0.12±6.44	2.32±10.36	−32.24±12.49**	−45.85±9.36***
β-mannosidase		4.31±14.17	4.39±18.9	−24.17±20.1*	−50.19±23**
β-galactosidase		−2.14±13.02	−14.36±15.33	−62.54±18.2**	−84.23±15.76***
GCase		−0.71±14.52	−60.93±15.21***	−93.43±4.72***	−97.93±2.98***
β-hexosaminidase		2.36±5.39	5.44±9.52	−21.73±20.08*	−35.38±18.48**
cathepsin D		0.98±9.73	−7.89±14.15	−33.78±22.66**	−41.94±21.6***
cathepsin E		−3.02±8.97	−5.33±9.23	−9.32±12.49	−12.81±11.32*

The percentage change of all enzymes activity tested for the pre-storage conditions is reported. Two different temperatures were tested: 4°C and room temperature (RT).

* = p<0.05;

**p<0.01;

***p<0.001.

### Freeze-thaw cycles

Five CSF samples were flash frozen in liquid nitrogen and stored at −80°C. The samples were thawed in ice altogether, assayed for the enzyme activities and frozen again at −80°C. Five cycles were performed using this procedure.

**Figure 2:**
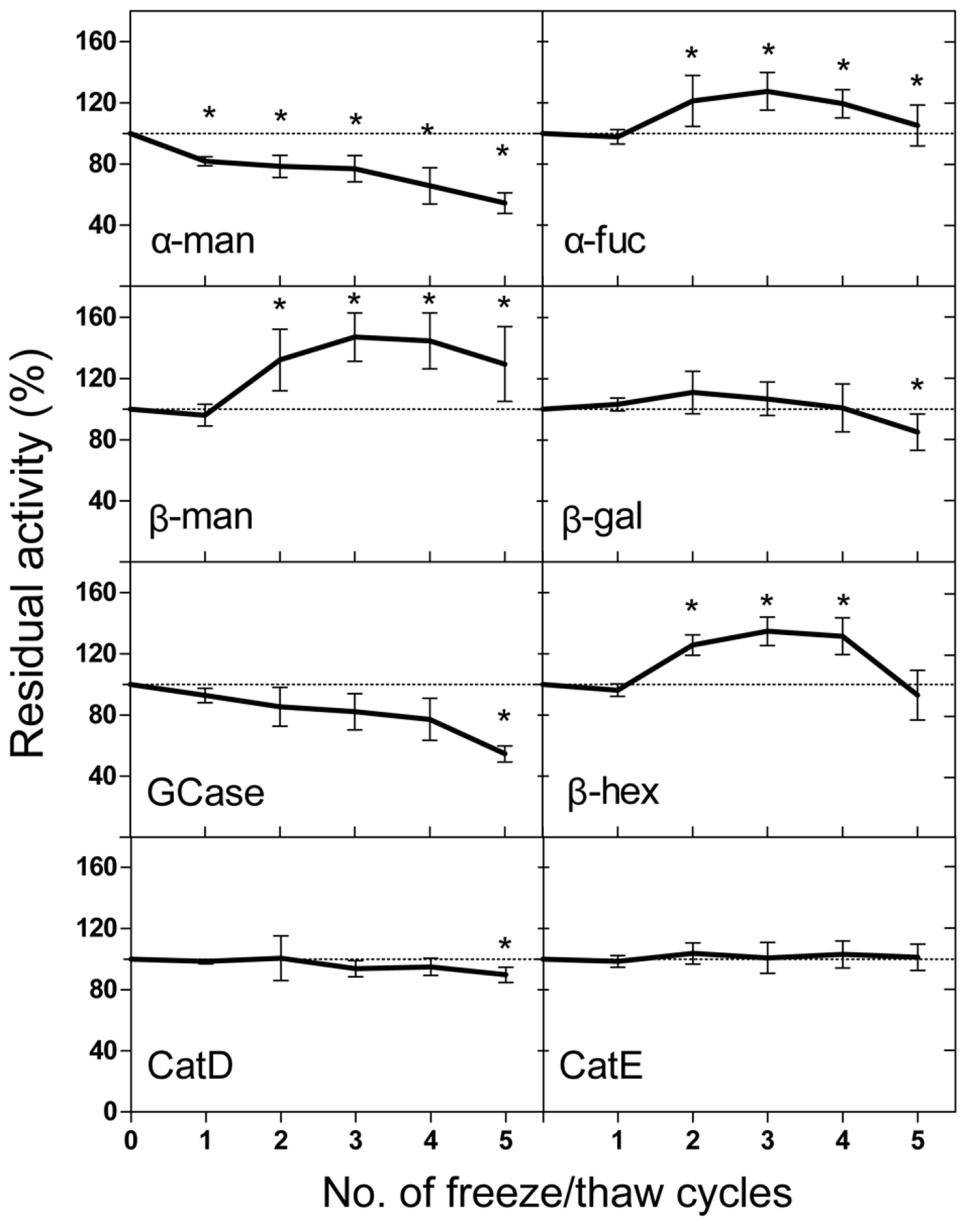
Freeze/thaw cycles. CSF samples were frozen in liquid nitrogen, thawed in ice, assayed for the activities of the lysosomal enzymes and frozen again. Five cycles were performed. The results are reported as the mean percentage change ± the SD of the activity with respect to fresh CSF. α-mannosidase; α–fuc: α-fucosidase; β-man: β-mannosidase; β-gal: β-galactosidase; GCase: β-glucocerebrosidase; β-hex: β-hexosaminidase; CatD: cathepsin D; CatE: cathepsin E.

The mean percentage activity changes of the lysosomal enzymes for up to 5 freeze and thaw (F/T) cycles with respect to fresh CSF are reported in Fig. 2 and Table 6. The cathepsins and β-galactosidase were the most stable enzymes, and remarkably, the cathepsin E activity was not influenced by the F/T cycles. The β-galactosidase and cathepsin D activities showed significant changes only after 5 cycles. The β-glucocerebrosidase activity showed a moderate decrease (−14.68±12.82%) after 2 cycles, while the activity significantly dropped after 5 cycles (−45.42±5.23%, p<0.01). The β-mannosidase, β-hexosaminidase and α-fucosidase activities significantly increased after 2 F/T cycles (by up to 120–140%, Table 6). The α-mannosidase activity decreased by −18.18±2.90% after the first cycle, and had a maximal decrease of −45.63±6.73% after 5 cycles (p<0.001).

**Table 6:** Relative percentage change of lysosomal enzymes activities during 5 freeze/thaw (F/T) cycles.

Enzyme	C1	C2	C3	C4	C5
α-mannosidase	−18.18±2.90**	−21.55±7.30**	−23.02±8.53**	−34.31±11.76***	−45.63±6.73***
α-fucosidase	−2.22±4.67	21.25±16.72*	27.51±12.29**	19.45±9.26*	5.13±13.31
β-mannosidase	−3.91±7.07	32.18±20.11**	47.18±15.83**	44.65±18.33**	29.45±24.55**
β-galactosidase	3.00±4.21	10.9±13.92	6.76±10.96	0.78±15.55	−15.02±11.77*
GCase	−7.3±4.64	−14.68±12.82	−17.93±11.79	−22.82±13.76	−45.42±5.23**
β-hexosaminidase	−3.85±3.94	25.65±6.68*	34.74±9.38**	31.39±12.02**	−6.98±16.16
cathepsin D	−1.55±1.46	0.59±14.63	−6.24±5.20	−5.06±5.66	−10.25±4.96*
cathepsin E	−1.51±3.88	3.82±6.96	0.85±10.12	3.13±8.80	1.16±8.53

The percentage change of all enzymes activity tested across five F/T cycles is reported.

* = p<0.05;

**p<0.01;

*** p<0.001.

### Effect of blood contamination

To test if a traumatic LP had a direct influence on the activities of lysosomal enzymes in CSF, three CSF samples were spiked with the blood of the same patient to obtain the following scalar erythrocyte concentrations: 50,000, 5,000, 1,000, 500 and 250 erythrocytes/µl. The spiked CSF samples were assayed for the different enzyme activities, and the results were compared with the respective neat sample.

**Figure 3:**
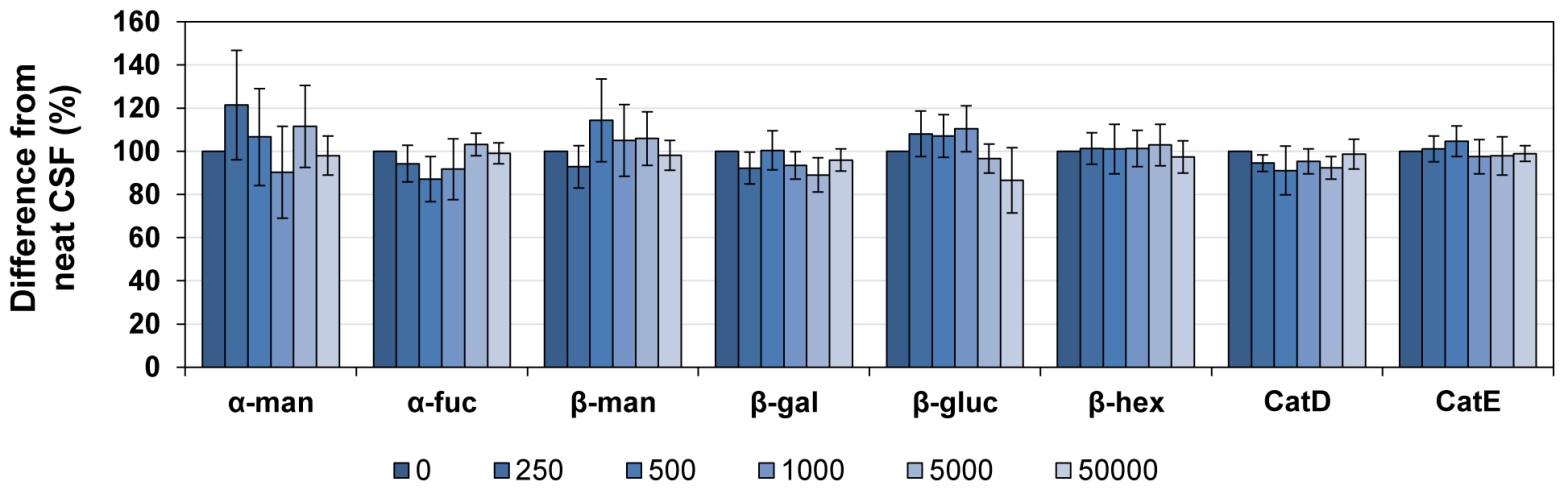
Effect of blood contamination on CSF lysosomal enzymes activities. CSF samples were spiked with whole blood to final concentrations of 250, 500, 1.000, 5.000, 50.000 erythrocytes/µl. Results are reported as percentage difference with respect to neat CSF (100%). Error bars represent standard deviation of % change.

Blood contamination of the CSF samples with up to 50,000 erythrocytes/µl did not significantly modify the activity levels of any of the lysosomal enzymes in a dosage-dependent way (Fig. 3).

### Longitudinal evaluation of the activities of lysosomal enzymes

Aliquots of 8 CSF samples were flash frozen and then stored at −20°C or −80°C. Four samples were used to assess the longitudinal stability of the enzymes at each temperature. One aliquot of each sample was thawed in ice and used for enzymatic assays after 1, 2, 3, 4, 8, 16, 32 and 40 weeks of storage. The differences between the enzyme activities of fresh CSF and the sample stored for 1 week were also calculated. To estimate the longitudinal stability of the activities of the enzymes, the samples stored for 1 week either at −20°C or −80°C were chosen as reference samples, and the percentage difference between each time point and the respective reference sample was calculated.

**Figure 4:**
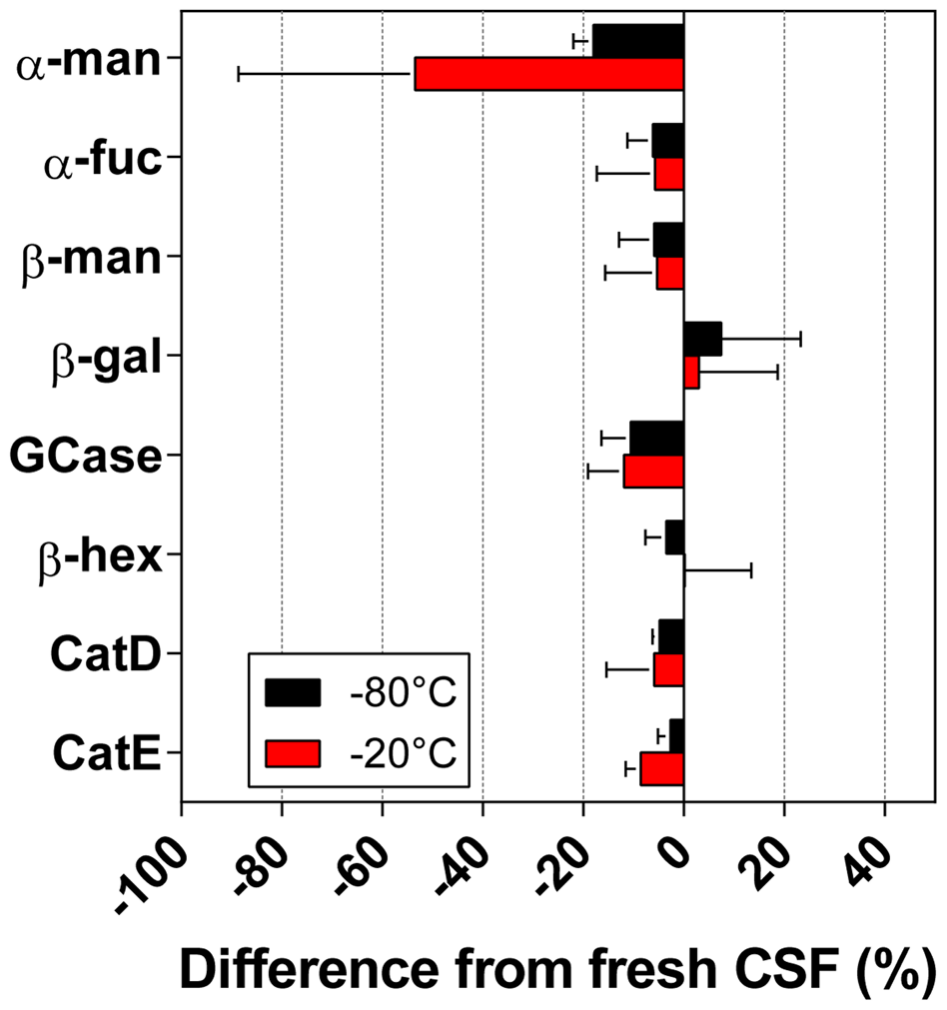
Effect of one week storage on lysosomal enzymes activities in CSF. Figure 4 depicts the percentage difference between fresh CSF and the same sample stored for 1 week either at −20°C or at −80°C. Data are presented as mean percentage change ± SD from fresh CSF.

Initially, the change in the activity percentage between fresh CSF and the same sample stored for 1 week at -80°C was calculated for each enzyme. The results are reported in Fig. 4. The α-mannosidase enzyme showed the most significant changes, with a reduction of −53.48±35.17% at −20°C and −17.94±4.03% at −80°C. The other enzymes showed changes between 2 and 10% when the samples that were stored for 1 week were compared to fresh CSF.

**Figure 5:**
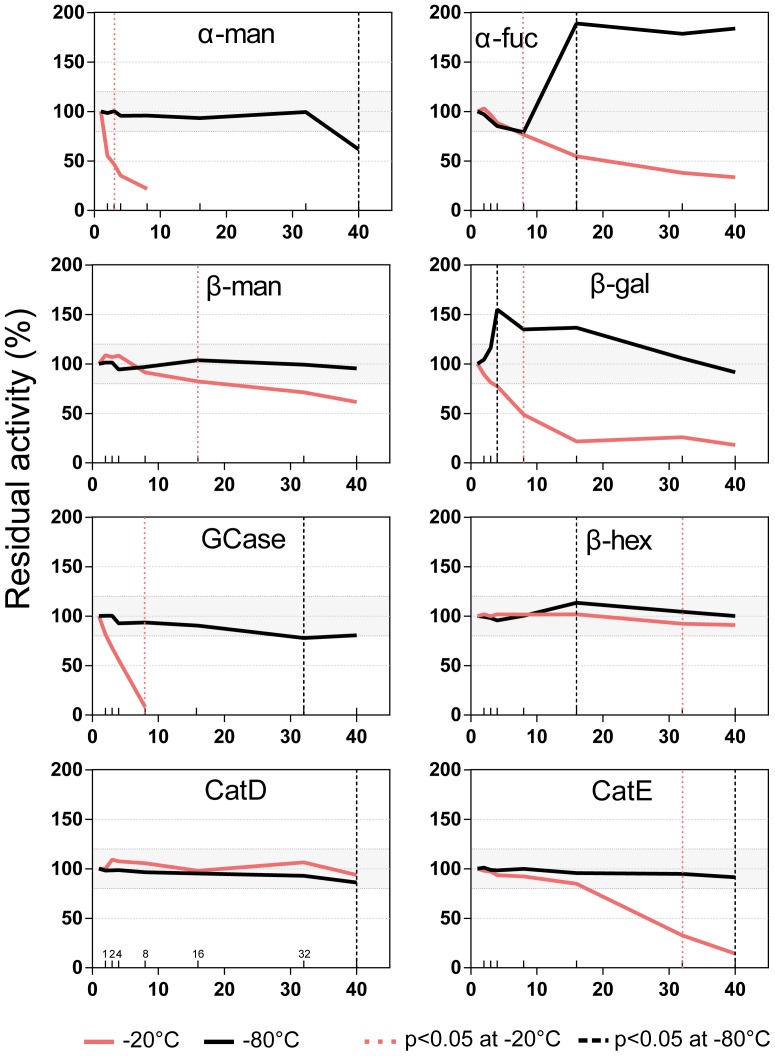
Longitudinal variability of the enzyme activities. Aliquots of the CSF samples were stored at −80°C or −20°C (solid lines) for up to 40 weeks. The results are reported as the mean percentage change with respect to the first week. The dotted lines mark the first time point at which the activity had a statistically significant change (p<0.05). The grey shadings depict ±20% variation. α-mannosidase; α–fuc: α-fucosidase; β-man: β-mannosidase; β-gal: β-galactosidase; GCase: β-glucocerebrosidase; β-hex: β-hexosaminidase; CatD: cathepsin D; CatE: cathepsin E.

The tested activities had different degrees of variability when assayed longitudinally. Fig. 5 shows the percentage changes of the activities of the tested enzymes for samples stored at −20°C or −80°C for a period of 40 weeks compared to the same sample after 1 week of storage (100%). Storage at −20°C was the most unstable freezing condition, with almost all of the enzymes showing a large reduction in their activities starting from the second week of storage. Only β-hexosaminidase and cathepsin D showed a high degree of stability at −20°C for up to 32 and 40 weeks, respectively (Fig. 5). Storage at −80°C allowed for more reliable measurements of the enzymatic activities. β-galactosidase and α-fucosidase were the most unstable enzymes when stored at −80°C, and they showed increases in their mean percentage activities of 54.9±38.08% after 4 weeks and 88.94%±36.19% after 16 weeks, respectively. The β-glucocerebrosidase activity change with respect to the first week of storage was not significant up to 32 week, when the mean percentage activity decreased by −22.36±18.18% (p<0.05). After 40 weeks of storage, β-glucocerebrosidase showed a mean percentage decrease of −19.60±19.11%. The CSF β-hexosaminidase activity had a significant increase after 16 weeks of storage with respect to the reference sample (13.55±8.56%, p<0.05). β-mannosidase, cathepsin D and cathepsin E did not show any significant changes up to 40 weeks of storage when stored at −80°C (Fig. 5 and Table 7).

**Table 7:** Relative percentage change of lysosomal enzymes activities for long term storage.

Enzyme	Storage T	w2(% mean ±SD)	w3(% mean ±SD)	w4(% mean ±SD)	w8(% mean ±SD)	w16(% mean ±SD)	w32(% mean ±SD)	w40(% mean ±SD)
α-mannosidase	−20°C	−44.89±17.58	−53.28±6.5	**−64.63±17.07** *	**−67.23±9.77** *	NA	NA	NA
α-fucosidase		3.05±9.07	−3.32±13.51	−11.27±15.11	**−23.41±3.5** *	**−45.2±10.39** ***	**−62.04±18.57** ***	**−66.37±16.69** ***
β-mannosidase		8.82±12.84	6.92±12.98	8.42±20.21	−8.6±13.53	**−17.65±16.31** **	**−28.75±10.96** ***	**−38.55±7.62** ***
β-galactosidase		−10.75±18.14	−18.67±16.1	−22.6±20.61	**−51.14±16.57** **	**−78.34±4.68** ***	**−74.14±21.12** ***	**−81.84±10.83** ***
GCase		−18.25±18.51	−32.15±28.33	−44.11±32.34	**−91.75±8.25** *	NA	NA	NA
β-hexosaminidase		1.76±1.22	−0.17±3.57	1.84±5.5	1.54±5.87	1.72±5.94	**−7.63±3.3** *	**−8.97±3.91** *
cathepsin D		0.12±5.7	9.28±8.76	7.49±6.33	5.58±6.41	−2.02±0.81	6.53±18.14	−6.35±2.15
cathepsin E		−1.98±4.55	−2.84±7.39	−6.45±4.16	−7.75±4.41	−15.22±21.91	**−67.19±24.29** **	**−85.74±11.54** **
α−mannosidase	−80°C	−1.62±8.57	0.35±4.93	−4.3±8.22	−4.23±11	−6.58±13.07	−0.6±17.64	**−38.27±17.25** *
α-fucosidase		−2.92±4.89	−8.98±9.04	−14.79±6.77	−20.88±14.26	**88.94±36.19** **	**78.54±36.67** **	**83.71±37.51** **
β-mannosidase		1.35±7.17	1.29±9.28	−5.69±8.18	−3.07±10.98	3.64±8.35	−0.66±8.73	−4.59±10.98
β-galactosidase		4.24±22.27	16.22±15.91	**54.9±38.08** *	34.76±31.06	36.59±42.14	5.55±20.49	−8.36±15.67
GCase		−0.10±5.85	−0.78±14.76	−7.30±4.05	−6.37±3.31	−9.38±5.58	**−22.36±18.18** *	−19.60±19.11
β-hexosaminidase		−0.73±2.8	−1.95±5.89	−4.39±5.13	0.38±4.19	**13.55±8.56** *	4.36±3.01	0.05±3.4
cathepsin D		−1.83±0.31	−1.55±5.51	−1.4±3.55	−3.53±1.3	−4.81±7.21	−7.13±12.11	**−13.89±13.7** *
cathepsin E		1.25±1.42	−1.22±2.44	−1.67±1.99	−0.02±2.59	−4.28±3.15	−5.14±4.68	**−8.62±8.21** **

Percentage changes of all enzymatic activities tested during longitudinal storage. Measurements were carried out up to 40 weeks either at −20°C or −80°C. T = temperature, w2 = 2^nd^ week, w3 = 3^rd^ week, w4 = 4^th^ week, w8 = 8^th^ week, w16 = 16^th^ week, w32 = 32th week, w40 = 40^th^ week.

* = p<0.05;

**p<0.01;

*** p<0.001;

NA = not available.

## Discussion

The potential diagnostic value of the activities of CSF lysosomal enzymes for different neurological diseases has been investigated since the end of the seventies (24, 25, 26, 27). Nevertheless, due to the thermal instability of these enzymes in biological fluids, this measurement has never been considered for clinical utilization (26, 28).

A renewed interest in these enzymes has emerged as a consequence of recent evidence highlighting the link between the impairment of the lysosomal system and PD pathology (11, 29, 30, 31). Interestingly, some recent studies have shown that the activities of some lysosomal enzymes in the CSF of patients with PD and other synucleinopathies is altered compared to control patients or patients with other neurodegenerative diseases (15, 16, 17, 18). Accordingly, the opportunity to use CSF lysosomal enzymes as biomarkers for PD deserves attention (32, 33).

Here, we systematically evaluated a series of pre-analytical factors that may affect the measurement of the activities of some lysosomal enzymes in CSF. Our results show that the total variability of these assays is minimally influenced by the within- and between-run variability, which ensures day-to-day repeatability. Other analytical conditions affected each enzyme in a different fashion.

The activities of some enzymes seem to be weakly influenced by pre-analytical factors. In particular, the activity levels of cathepsins D and E showed elevated resilience to F/T cycles, high longitudinal stability up to 40 weeks and no significant change when kept at 4°C for at least 24 hours before freezing. Accordingly, the measurement of their activities in CSF may represent a promising biomarker when also considering their possible involvement in the pathogenesis of PD and AD (17, 34, 35). To our knowledge, this is the first study assessing the longitudinal stability of the cathepsin D and E activities in CSF. The β-mannosidase activity was found to be stable when the CSF samples were kept at 4°C for up to 24 hours and then stored at −80°C for up to 40 weeks similarly to the cathepsins, but its activity has a marked fluctuation after F/T cycles. Among the other lysosomal enzymes, the α-mannosidase activity was altered by almost all of the pre-analytical factors tested. For instance, after only 1 week of storage the α-mannosidase activity showed a large decrease (∼20% at −80°C and ∼60% at −20°C), which confirmed the findings of a previous study (23). The β-hexosaminidase activity also showed marked fluctuations after F/T cycles. The β-galactosidase and α-fucosidase activities had similar inconsistencies when evaluated longitudinally. These changes could be explained by the presence of unstable enzymatic modulators in the CSF (36). Moreover, some enzymes are part of multi-enzyme complexes that could be disjointed during freezing steps, which could increase the affinity of each enzyme to the synthetic substrate (37).

Our results also showed that the CSF β-glucocerebrosidase activity is influenced by pre-analytical factors. A 40% decrease of the CSF β-glucocerebrosidase activity was reported when the samples were directly frozen at −80°C with respect to samples snap-frozen in liquid nitrogen (23). In this study, we were not able to find significant difference in the CSF β-glucocerebrosidase activity between the two conditions after 1 week of storage (same time frame as in the previous study). Furthermore, at −80°C, β-glucocerebrosidase was stable for up to 32 weeks. These differences may be due to the time-delay and the storage temperature before freezing, which were not specified in the previous study and significantly affect the CSF β-glucocerebrosidase activity. Moreover, the two studies are not directly comparable because in Goi et al., the β-glucocerebrosidase enzymatic assay was performed in absence of TDC. Taurodeoxicolic acid enhances the specificity of the assay inactivating specific isoenzymes, as it has been shown in leucocytes (38).

The final recommendations for the proper assessment of the activities of CSF lysosomal enzymes emerging from this work are: i) the CSF samples should be centrifuged and kept at 4°C immediately after the LP and stored at −80°C within 1 h of the collection; ii) the β-mannosidase, cathepsin D and cathepsin E activities should be tested within 40 weeks of the LP, and the GCase activity should be tested within 32 weeks; and iii) the number of F/T cycles do not significantly affect cathepsins D and E for up to four cycles, but they should be avoided for β-glucocerebrosidase and the other enzymes.

In conclusion, we have shown that after applying the correct pre-analytical operating procedures, the measurement of β-mannosidase, β-glucocerebrosidase, cathepsin D and cathepsin E in CSF can be reliable and reproducible. These conditions are mandatory to correctly compare the results from different cohorts aimed at confirming the role of the activities of CSF lysosomal enzymes as potential biomarkers for neurodegenerative disorders, with a special interest on Parkinson's disease.
