# Correction: Factors Influencing the Measurement of Lysosomal Enzymes Activity in Human Cerebrospinal Fluid

**DOI:** 10.1371/journal.pone.0114809

**Published:** 2014-11-25

**Authors:** The PLOS ONE Staff

The tenth author’s name is spelled incorrectly. The correct name is: Omar M.A. El-Agnaf.

## References

[1] Persichetti E, Chiasserini D, Parnetti L, Eusebi P, Paciotti S, et al (2014) Factors Influencing the Measurement of Lysosomal Enzymes Activity in Human Cerebrospinal Fluid. PLoS ONE 9(7): e101453 doi:10.1371/journal.pone.0101453 24983953 10.1371/journal.pone.0101453 PMC4077821

